# *In Vitro* Micropropagation of Industrially and Medicinally Useful Plant *Aloe trichosantha* Berger Using Offshoot Cuttings

**DOI:** 10.1155/2020/3947162

**Published:** 2020-07-16

**Authors:** Atsbeha Hailu, Desta Berhe Sbhatu, Haftom Baraki Abraha

**Affiliations:** ^1^College of Natural and Computational Sciences, Mekelle University, P.O. Box 231, Mekelle, Ethiopia; ^2^Mekelle Institute of Technology, Mekelle University, P.O. Box 1632, Mekelle, Ethiopia

## Abstract

This study was aimed to develop *in vitro* micropropagation protocol of *Aloe trichosantha* Berger using offshoots as explants. MS media supplemented with plant growth regulators helped explants develop shoots within about 14 to 17 days. The mean number of days to shooting has decreased from 16.8 ± 0.8 with 0.5/0.5 mg/L BAP/NAA supplement to 15.5 ± 0.5 with 2.0/0.5 mg/L BAP/NAA. While the mean shoot number has increased with increasing the concentration of BAP supplements, the reverse was true with mean shoot lengths, whereas supplement of 2.0/0.5 mg/L BAP/NAA has generated significantly more shoots (17 ± 3.8), and longer shoots were produced with the addition of 0.5/0.5 and 1.0/0.5 mg/L BAP/NAA. In regard to rooting, though higher concentrations of NAA have resulted in quick rooting, the rooting performance in terms of mean number and length of roots was better with low concentrations. All the plantlets subjected to greenhouse acclimatization in cocopeat have survived. Secondary acclimatization in composted and manured soil media has also resulted in 93 to 95% survival rate. Lighting conditions (nursery shade or direct sunlight) of secondary acclimatization did not lead to any difference in the survival rate of the plantlets.

## 1. Introduction

*Aloe* L. (Aloaceae) comprises about 450 species of flowering plants [1–3]. Some records in the Kew Botanic Garden put the number to more than 500 [4]. They are easily recognized by their rosettes of large, thick, succulent leaves that are sometimes spotted with their leaf margins almost always armed with sharp teeth [1]. Aloes are distributed in almost all parts of the world and are represented in the key biodiversity hot spots including the Horn of Africa, Madagascar, and Indian Ocean islands. Aloes are mainly found in the southern and eastern African regions [2]. Most of them are adapted to extreme environments and demonstrate strong resistance to drought, salty, or poor soils. In the flora of Ethiopia and Eritrea, they stretch from sea level at Massawa (*A. eumassawana* Carter, Gilbert and Sebsebe) to about 3,500 meters at Ankober (*A. ankoberensis* Gilbert and Sebsebe), showing high degree of endemism [1].

Aloes include many multipurpose species with ecological and economical significance. Many species of aloe (especially *A. vera* L.) are exploited in the cosmetic, medical, pharmaceutical, and beverage industries, while many others are extremely valuable for their ecological significance. Moreover, nearly all aloes have been used for medicinal purposes for ages to treat many human and animal (livestock) ailments [5–7]. The gels and latexes of aloes are used for healing wounds and burns, anesthetizing tissues, stopping or slowing fungal, viral, and bacterial growths, improving blood flow, and acting as inflammatory, antiaging, and antiallergic agents [5, 8, 9]. Studies revealed that leaf gels and latexes of aloes contain amino acids and proteins, enzymes, minerals, vitamins, polysaccharides, and biological stimulators [10].

Generally speaking, aloes have restricted geographical and ecological distribution, existing in small populations [1]. Therefore, many species are now threatened because of habitat destruction caused by global and local anthropogenic factors. Change in land use in many places can wipe out entire natural stands altogether. Likewise, some species can also be excessively exploited for traditional medicine and commercial purposes. In fact, Demissew and Nordal [1] ascertained that all species of *Aloe* (except *Aloe vera* L.) are listed in the CITES (Convention on the International Trade in Endangered Species of Wild Fauna and Flora) Appendix II due to their vulnerability. This listing forbids the use of any plant species in wild stands for industrial purposes. Ethiopian and Eritrean aloes cannot be exceptions.

These imply that exploitation of aloes for industrial purposes requires the cultivation of the plants at commercial scale. The sexual reproduction of aloes is ineffective due to male sterility. Thus, they propagate primarily by means of sucker or offshoots [7, 11, 12]. Unfortunately, aloes' natural vegetative propagation is, likewise, very slow and ineffective to meet any demand of high-quality planting material for commercial cultivation [6]. *A. trichosantha*, belonging to the Tigrai and Eritrea floristic regions of the flora of Ethiopia and Eritrea, is one of the species being exploited for industrial purposes in Ethiopia. An Ethiopian company, specialized in producing jute bags for packing coffee, cereals, and oil seeds was granted to harvest *A. debrana* and *A. trichosantha* subsp. *longiflora* leaves from some natural stands in North Central Ethiopia until it gets into some commercial cultivation [13]. The leaves are processed to produce extracts used in treating the bags before they are used for packaging coffee. The species is also the source of important compounds that inhibit the growth of many bacterial and fungal pathogens [14]. Hence, efforts for commercial cultivation of the plant are necessary. Large-scale micropropagation of aloes using vegetative explants has been proved successful in many species and varieties [15–21]. This article reports the findings of a study carried out to develop suitable and reproducible protocol for *in vitro* regeneration of *A. trichosantha*. It has attempted to find out the optimal concentrations and combinations of plant growth regulators (PGRs) for *in vitro* establishment, shoot production, and rooting, and to assess the acclimatization of *in vitro* plantlets under different acclimatization conditions.

## 2. Materials and Methods

### 2.1. Location of Research Site

The study was conducted in the Tigrai Biotechnology Center Pvt. Ltd. Co. Lab, formerly known as the Mekelle Plant Tissue Culture Laboratory. The facility is located in Mekelle, Tigrai, Ethiopia (alt.: 1979 masl; lat.: 13° 30″ 0′ N; long.: 39° 28″ 11′ E), about 200 km southeast of the historic city of Aksum. The mean annual temperature of Mekelle ranges from 16°C to 20°C.

### 2.2. Collection and Sterilization of Explants

**Figure 1:**
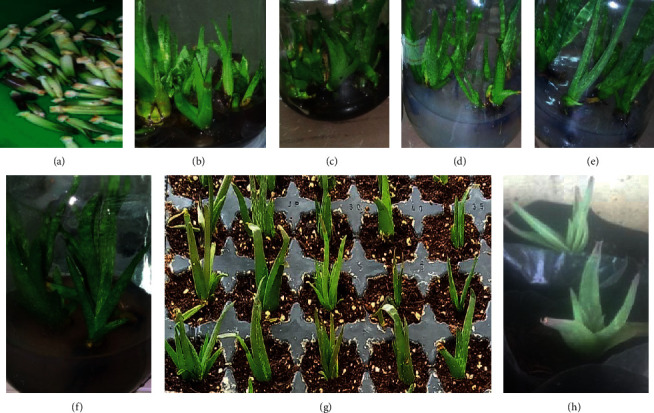
*In vitro* micropropagation of *A. trichosantha*. (a) Cleaned explants; (b) regenerating (shooting) explants cultured in an MS medium enriched with 1.5/0.5 mg/L BAP/NAA; (c) regenerating explants cultured in an MS medium enriched with 2.0/0.5 mg/L BAP/NAA; (d) and (e) rooting shoots cultured in an MS medium enriched with 1.0 mg/L NAA; (f) rooting shoots cultured in MS media enriched with 1.5 mg/L NAA; (g) *ex vitro* plantlets after two weeks of planting in cocopeat in greenhouse; (h) *ex vitro* plantlets after two weeks of planting in a composted soil medium.

Offshoots of *A. trichosantha* were collected from wild stands at northwestern outskirts of Mekelle City, Ethiopia. Healthy and vigorous mother plants with miniature offshoots were identified in the collection site. Then, 60 to 70 offshoots were collected from the bases of the mother plants aseptically and gently with minimum physical damage and contamination. The offshoots were readied for sterilization by removing their outer and older minileaves and trimming them into 2-3 cm long explants. Sterilization began by thoroughly washing the explants in running tap water for 30 minutes to remove debris off the explants. The explants were immersed in Tween-20 and soap solution in an Erlenmeyer flask and were shaken for about 5 more minutes. Then, they were washed with distilled water to remove traces of detergents, soaked in 0.25% Kocide (active ingredient: cupper hydroxide), 0.25% Ridomil (active ingredient: metalaxyl-m), and 0.25% Bayleton (active ingredient: triadimefon 50%) by shaking the flask in clockwise and anticlockwise directions for 10 minutes, rinsed gently three times with distilled water to remove any traces of chemicals, soaked in 5% v/v of NaOCl while shaking for about 10 minutes, and rinsing them with sterilized distilled water to remove traces of NaOCl. Finally, the explants were readied for sterilization under the laminar air flow cabinet by soaking and shaking them in fresh soap solution composed of sterile distilled water, 0.25% w/v HgCl_2_ aqueous solution, and 2 drops of Tween-20, and rinsing them three times with sterile distilled water to remove traces of HgCl_2_ (Figure 1(a)). Parts of the explants damaged by sterilization were removed using a sterile scalpel blade to produce turgid explants ready for incubation [22–27].

### 2.3. Preparation of Growth Media

Growth media were prepared according to the procedure of Murashige and Skoog (MS) [28]. Benzylaminopurine (BAP) and naphthaleneacetic acid (NAA) were the PGRs chosen to enrich the MS media. Full-strength MS media were used in the study of initiation and shooting, and half-strength media were used to study rooting. The growth media were prepared at a rate of 1.0 L per formulation. The formulation of each growth medium was prepared by placing a 1.0 L beaker holding 500 mL of sterile distilled water on a magnetic stirrer. Then, 100 mL macronutrient, 100 mL micronutrient, 10 mL organic supplement solutions, the required amount of the PGR, and 30 gm/L sucrose (C-source) were mixed and homogenized. Thereafter, sterile distilled water was added until the growth medium reaches 1.0 L mark and the pH of solution was adjusted to 5.8 by adding 1N NaOH and 1N HCl as appropriate before adding 8 g agar for gelling, thus producing nutrient agar media. The growth media were autoclaved at 121°C and 15 psi pressure for 20 minutes and cooled to 60°C. Finally, 40 mL of the content from each formulation was dispensed into 300 mL labeled magenta culture bottles and kept for a week at room temperature for inspecting their sterility.

### 2.4. Micropropagation Experiments

Shoot initiation was carried out by incubating sterilized and live explants (*n* = 50) in full-strength MS media enriched with 0.5/0.5 mg/L BAP/NAA in 300 mL magenta bottles. The explants were cultured in a growth room rack for eight days at a temperature of 25 ± 0.5°C under a fluorescent tube light with a 16-hour photoperiod and light intensity of 2000–2500 lux [27]. Then, healthy, live, and clean explants were harvested and transferred to shooting (regeneration) media. Full-strength MS media enriched with different concentrations of BAP in combination with constant concentration NAA (i.e., 0/0, 0.5/0.5, 1.0/0.5, 1.5/0.5, and 2.0/0.5 mg/L BAP/NAA) were readied for shooting. Two explants were inoculated into each treatment, and all treatments were replicated four times. Then, they were incubated in growth racks for four weeks in similar conditions set for initiation. The explants were transferred to fresh shooting media to minimize damage due to phenol accumulation and were kept for three more weeks to produce 3–4 cm shoots for rooting. Rooting experiment was carried out in half-strength MS media enriched with four different concentrations (0.25, 0.50, 1.0, and 1.5 mg/L) of NAA filled in 300 mL magenta culture bottles. One unsupplemented MS medium was added as control. Each treatment was inoculated with two shoots and replicated seven times. The shoots were incubated in growth rooms for four weeks in similar conditions set for initiation to produce ≥ 5 cm plantlets (i.e., rooted shoots).

### 2.5. Acclimatization of Plantlets

Plantlets were gently removed from the rooting media in the magenta culture bottles and were washed in running tap water to remove traces of agar. Then, the plantlets were soaked in hot water (around 40°C) for about 5 minutes to remove any oily stuff that would hamper the absorption capacity of the roots of plantlets. Plantlets with well-developed roots were prepared for acclimatization. The acclimatization study comprises primary (greenhouse) and secondary (nursery shade and open field) acclimatization. Greenhouse acclimatization was carried out in cocopeat filled in a protray. Plantlets were planted in cocopeat in the protray and were kept in greenhouse for three weeks. The greenhouse was adjusted to progress from high relative humidity (80–90), low temperature (25 ± 2°C), and low light intensity (1,200 lux), through medium relative humidity (70–80), medium temperature (26 ± 2°C), and medium light intensity (2,500 lux) to low relative humidity (60–70), high temperature (27 ± 2°C), and high light intensity (5,000 lux). Then, the plantlets were readied for secondary acclimatization in composted (sand, soil, and compost at 1 : 1 : 1 ratio) and manured (sand, soil, and manure at 1 : 1 : 1 ratio) soil media filled in polyethylene bags (length: 15 cm; dia.: 9 cm) under nursery shade and in direct sunlight. The plantlets were kept for four weeks. They were watered daily and closely studied.

### 2.6. Data Collection and Analyses

The initiation experiment has yielded live and healthy explants. The shooting study has yielded 3–4 cm shoots. The rooting study has yielded rooted shoots (explants). The acclimatization studies have yielded survival rates. Therefore, the quantitative data out of these studies were rate of survival to initiation, days to shooting and rooting, number of shoots and roots, length of shoots and roots, and rate of survival to acclimatization. Data of days to shooting and rooting were collected by inspecting the incubated plantlets every two days. Data on the number and length of shoots and roots were collected after the end of the shooting and rooting studies, respectively. Then, collected data were organized and analyzed using descriptive and inferential statistical methods using the statistical package for social science (SPSS version 20). All comparisons were made at *a priori* established significance level of *p* ≤ 0.05. Qualitative observations were also made to provide better insights about measurements and recordings of the observations.

## 3. Results and Discussion

### 3.1. Initiation of Explants

Culturing explants in an initiating medium are a customary practice of *in vitro* plant tissue culture and micropropagation. Combinations of BAP and NAA at different concentrations are often used to initiate explants before incubated in shooting, regeneration, or micropropagation media. This procedure yields clean and live explants, uncontaminated and with no sign of rotting [22, 25, 28]. The explants are believed to have some vital activities leading to limited growth. *A. trichosantha* explants (*n* = 50) cultured in 0.5/0.5 mg/L BAP/NAA for eight days have yielded 96% clean and viable explants. Supplementing the initiation medium with PGRs that contain 1 : 1 ratio of cytokinin (BAP) to auxin (NAA) promotes quick cell division in the explants without differentiation, which can lead to callusing if the duration of culturing is increased [29, 30]. Therefore, quickly initiated explants become convenient for shooting treatment. Many studies have been conducted with combinations of low concentrations of BAP and NAA (i.e., 0.10 to 0.50 mg/L) to initiate explants with outstanding results. Initiation of *A. elegans* Todaro with 0.25/0.10 mg/L BAP/NAA [31], *A. adigratana* Reynolds with 0.50/0.50 mg/L BAP/NAA [32], and *A. percrassa* Todaro with 0.20/0.20 BAP/NAA [22] produced 95 to 100% clean and viable explants. In fact, many of the viable explants of *A. percrassa* developed one to four shoots in four weeks. The use of BAP/NAA at 0.10 to 0.50 mg/L for initiating *A. vera* L. explants has yielded 100% clean and viable explants with some degree of shoot formation within one to four weeks as observed by several researchers [11, 19, 33, 34]. The quick emergence of shoots at the initiation stage is generally accountable to higher content of PGRs in the explants.

### 3.2. Shoot Performance

Shooting was operationally defined as the development and growth of one or more new shoots from the base of an initiated explant (i.e., viable explant). The shooting performance of the plant was evaluated through the analyses of days to shooting, number of shoots per explant, and length of shoots (in cm) as the function of the application of PGRs. The data on these parameters were analyzed using analysis of variance (ANOVA). The results have shown that the mean days to shooting, number of shoots, and length of shoots were statistically significantly different among the treatments (Table 1).

Cultured plantlets were visually inspected every two days to observe any emergence of shoots. As shown in Table 1, all explants in all the treatments have developed shoots within about 14 to 17 days (Figure 1(b) and 1(c)). Swollen adventitious buds were observed at the bases of the explants before the emergence of the shoots. However, the explants did not yield shoots till the end of the second week. Though not much profound, the mean number of days to shooting decreases from 16.8 ± 0.8 with 0.5/0.5 mg/L BAP/NAA supplement to 15.5 ± 0.5 with 2.0/0.5 mg/L BAP/NAA. The use of BAP and NAA to enrich shoot proliferation MS agar media is critical in many species such as *A. adigratana* [32], *A. barbadensis* [35], *A. elegans* [31], *A. polyphylla* [36], and *A. vera* [11].

The shooting experiment yielded shoots in all PGR-supplemented treatments in four weeks. However, some accumulation of phenol was observed. Thus, all newly produced shoots were separated from the explants and transferred into fresh PGR-free media for additional three-week culturing. The subculturing of the shoots into fresh PGR-free media mitigated the problem of phenol accumulation. Three observations were made from the shooting experiment: (a) the significantly highest mean shoot number (17.3 ± 3.8) was recorded in MS media enriched with the high concentration of BAP (2.0 mg/L); (b) the mean number of shoots per explant tended to increase with increasing the concentration of BAP from 0.50 to 2.0 mg/L; and (c) unsupplemented MS media (control) did not produce any shoots after seven weeks of subculturing. MS media enriched with 1.5/0.5 mg/L BAP/NAA have also yielded a high mean shoot number (9.3 ± 1.6).

Many researchers reported the absence of shooting in explants of *A. vera* genotypes cultured in MS shooting media without PGR supplements [11, 19, 37]. Likewise, Gupta et al. [11] and Baksha et al. [35] observed maximum shoot regeneration in MS media enriched with 2.0/0.5 mg/L BAP/NAA. Baksha and co-workers [35] reported that explants enriched with 2.0/0.5 mg/L BAP/NAA have produced 10 ± 0.50 shoots while other treatments with higher BAP produced up to 22.16 ± 3.24 shoots per explant. Also, Biswas et al. [33] observed that 2.0/0.5 mg/L BAP/NAA results in the best response to *A. indica* L. Similarly, Saggoo and Kaur [38] suggested that MS media enriched with 2.0/0.3 mg/L BAP NAA are best for micropropagation of *A. vera*. Studies conducted by Nayanakantha et al. [37] indicated that a medium containing 4.0 mg/L BAP + 0.20 mg/L + 1.0 g/L PVP + 10.0 mg/L citric acid is best for shooting, which produced 18.7 ± 1.2 shoots per explant. Furthermore, Khanam and Sharma [19] reported that the MS medium enriched with 4.0/0.2 mg/L BAP/NAA to be best on *A. vera* in terms producing more shoots.

Our study has shown that the mean length of shoots (in cm) observed in the treatments that yielded more shoots is low. Since the amount of nutrient in all the treatments is the same, treatments producing more shoots will naturally have smaller sizes while those that are fewer will have bigger sizes. However, this logical inference has to be considered with caution because that proportionality often disappears when the mean values (of shoot number) are optimum (4 to 9) and their differences are smaller. Anyway, we have observed that all PGR supplementations produced 2.5–5.5 cm long shoots in seven weeks. Several studies reported similar results with similar PGR supplements [11, 22, 35, 39].

### 3.3. Rooting Performance

Evaluation of the rooting performance of *A. trichosantha* shoots was made based on the data of days to rooting, number of roots, and length of roots (cm). Results of ANOVA have shown that the mean days to rooting, number of roots, and length of roots were statistically significantly different among the treatments (Table 2; Figures 1(d)–1(f)).

*A. trichosantha* shoots could root in 8 to 17 days after incubation in half-strength MS media enriched with NAA at 0.25 to 1.5 mg/L. As the concentration of the NAA supplements increased from 0.25 mg/L to 1.5 mg/L, the mean number of days to root to decrease sharply by about half. Shoots incubated with no PGR supplements have failed to produce roots by the end of the third week of culturing. Some callus like, yellowish structures were observed at the bases of the shoots. NAA is one the most preferred PGRs for rooting of shoots through *in vitro* micropropagation. Compared to other Ethiopian aloes whose micropropagation capacities are studied, the rooting response of *A. trichosantha* is relatively quicker. The rooting responses of *A. adigratana* (15 to 24 days) [32], *A. elegans* (15 to 20 days) [31], and *A. percrassa* (15 to 17) [22] are slower by about one week. Moreover, several researchers have reported slower rooting responses in some genotypes of *A. vera* (23 to 28 days) [11] and *A. barbadenesis* (14 to 19 days) [40, 41].

The rooting performance in terms of the mean number of roots per explant, however, was better in the MS medium enriched with lower concentration (0.25 to 1.0 mg/L) of NAA. Even though the 1.5 mg/L led to the quick emergence of roots, the roots remain to be significantly fewer as compared to those in the other treatments, in general, and to the 1.0 mg/L NAA enriched treatment, in particular. Interestingly, raising the concentration of NAA to 1.5 mg/L and beyond produces a lower mean root number compared to lower concentrations in *A. barbadensis* [40], *A. elegans* [31], *A. percrassa* [22], *A. polyphylla* [36], *A. indica* [33], and *A. vera* [42, 43]. The NAA supplements also resulted in statistically different mean root length (cm) among the treatments. The treatment enriched with lowest concentration of NAA (i.e., 0.25 mg/L) yielded statistically highest mean root length. The values of the mean root length tend to decrease with increasing NAA concentration. Similar patterns were observed many species including *A. adigratana* [32], *A. elegans* [31], *A. percrassa* [22], *A. polyphylla* [36], and *A. vera* [42].

### 3.4. Acclimatization of Plantlets

Acclimatization of *in vitro* generated plantlets is the most critical step in plant tissue culture and micropropagation. Plantlets with similar sizes of roots and shoots (*n* = 70) were harvested from the rooting media, planted in cocopeat filled in a protray, and were placed in the greenhouse for three weeks for primary acclimatization. All (100%) of the plantlets have survived (Table 3; Figure 1(g)). As we had observed in *A. adigratana*, *A. elegans*, and *A. percrassa*, aloe plantlets can only fail to thrive if and only if they are exposed to contaminants that make them to rot. Thus, many researchers observed 90 to 100% survival rate in many species of *Aloe* after primary acclimatization [22, 31, 32, 36, 44–46].

A secondary acclimatization study was carried out for four weeks in nursery shade and direct sunlight with composted and manured soil as a planting medium. Only one plantlet has died from each group due to rotting yielding 93 to 95% survival rate (Table 3, Figure 1(h)). The plantlets have grown by exhibiting similar looks as the mother plants. High output of primary acclimatization leads to high plantlet survival after secondary acclimatization. However, care has to be made to avoid or minimize any plantlet damage that exposes them to contaminants or leads them to lose their vascular contents through drainage or evaporation. The rotting and death of the explants reported in the works of Abraha et al. [22], Niguse [32], and Welehaweria [31] took place very quickly. Had the death of the plantlets been linked to environmental stress, more plantlets could have been died from the plantlets acclimatized in direct sunlight.

## 4. Concluding Remarks

We described the micropropagation protocol of the plant. We showed that the plant can easily and quickly be propagated *in vitro* with the application of PGRs. Explants were easily initiated in a basal MS medium supplemented with equal proportion of BAP and NAA. Generally speaking, increasing the concentration, the tested PGRs enhanced the regeneration and rooting response of the plant. Future studies may focus on refining the sterilization, initiation, regeneration (shooting), rooting, and acclimatization procedures. *In vitro* micropropagation studies can be done with many formulations and combinations of PGRs under more refined concentrations and various sources of explants. Furthermore, the plant's phytochemistry, medicinal importance, and ecological significance need to be explored.

## Figures and Tables

**Table 1:** Effect of PGRs with different concentrations and combinations of BAP and NAA on shooting responses of *A. trichosantha* Berger.

PGRs (mg/L)		Mean (SD) values		
BAP	NAA	Days to shooting	Shoots per explant	Shoot length (cm)
0.00	0.00	0	0	0
0.50	0.50	16.8 (0.8)^a^	5.5 (2.0)^c^	4.8 (0.8)^a^
1.00	0.50	16.3 (0.4)^ab^	4.8 (2.3)^c^	4.6 (0.4)^a^
1.50	0.50	16.0 (0.5)^ab^	9.3 (1.6)^b^	3.1 (0.6)^b^
2.00	0.50	15.0 (0.5)^b^	17.3 (3.8)^a^	3.3 (0.4)^b^

Mean		12.90	7.35	3.15
CV%		4.85	45.45	20.54
LSD		0.96	5.15	0.99

Means in the same column with different letters are statistically significantly different at *p* ≤ 0.05; CV: coefficient of variance (%); LSD: least significant different.

**Table 2:** Effect of different concentrations of NAA on rooting responses of *A. trichosantha* Berger.

PGRs	Concentration (mg/L)	Mean (SD) values		
		Days to rooting	Root number	Root length (cm)
Control	0.00	0	0	0
NAA	0.25	15.7 (1.2)^a^	7.2 (1.8)^a^	3.9 (0.5)^a^
	0.50	13.6 (2.5)^b^	7.9 (2.7)^a^	2.7 (0.9)^b^
	1.00	9.2 (1.0)^c^	8.0 (1.4)^a^	2.1 (0.3)^b^
	1.50	9.7 (0.9)^c^	4.2 (0.8)^b^	2.2 (0.5)^b^

Mean		9.63	5.43	2.17
CV%		15.12	29.60	33.70
LSD		1.61	1.77	0.81

Means in the same column with different letters are statistically significantly different at *p* ≤ 0.05; CV: coefficient of variance (%); LSD: least significant different.

**Table 3:** Survival of plantlets of *A. trichosantha* Berger three weeks after planting.

Acclimatization media	Light condition	Quantity	Survival (%)
Artificial media (100% cocopeat)	Greenhouse	70	100
Composted media (soil, sand, compost at 1 : 1 : 1)	Nursery shade	20	95.0
	Direct sunlight	20	95.0
Manured media (soil, sand, manure at 1 : 1 : 1)	Nursery shade	15	93.3
	Direct sunlight	15	93.3

## Data Availability

The datasets used and/or analyzed during the current study are available from the corresponding author on reasonable request.
